# Examination of American Canned Meats in Germany

**Published:** 1896-11

**Authors:** 


### EDITORIAL.

### THE EXAMINATION OF AMERICAN CANNED MEATS IN GERMANY.

And now the canned meats which we are shipping to Germany must be opened and examined before they can be offered for sale, which practically means their exclusion from that country. Is it not time for our government to take some step that will savor of retaliation against these prohibitive measures aimed at American products? Why not, for a starter, prohibit our Sugar Trust from pouring millions annually into Germany for the purchase of sugar, the beet-roots for which could be grown better and in larger crops on thousands of acres of our own lands that are impoverishing the farmers in their endeavor to raise cereals? This would be of twofold value to our people: It would save millions to our own people, and would restrict the power of the Sugar Trust; for, with the growth of sugar-beets independent refineries would spring up over our land, and a single concern could no longer control the whole output of sugar and be the sole employer of a very large body of people, whereby the trust is enabled to fix the terms of employment and wages, from which there is no appeal. Let us give it a trial, for these every-day restrictions placed by foreign nations on our products are gradually lessening the foreign field for our surplus animal food-products, and thus contributing to the continued depression of our live-stock industry. This comes directly home to the veterinary profession, and we should be quick to respond, and by our influence aid the accomplishment of this much-to-be-desired state of affairs. Move on!

